# Two culture approaches used to determine the co-composting stages by assess of the total microflora changes during sewage sludge and date palm waste co-composting

**DOI:** 10.1186/s40201-014-0132-4

**Published:** 2014-11-15

**Authors:** Loubna El Fels, Fatima-Zahra El Ouaqoudi, Farid Barje, Mohamed Hafidi, Yedir Ouhdouch

**Affiliations:** Laboratory of Ecology and Environment (L2E) (Unit Associated with the CNRST, URAC32), Faculty of Science Semlalia, Cadi Ayyad University, BP: 2390 Marrakech, Morocco; Laboratory of Biology and Biotechnology of Microorganisms, Faculty of Science Semlalia, Cadi Ayyad University, BP: 2390 Marrakech, Morocco

**Keywords:** Sludge, Composting time extract agar, Standard media, Microbial succession

## Abstract

Indigenous microflora community changes during six months of co-composting of activated sewage sludge and date palm waste was investigated using two different culture approaches. In order to evaluate the co-composting process evolution for mixture A and B, growth standard media (GSM) and Compost Time Extract Agar (CTEA) are used. Enumeration for indigenous flora abundance on GSM medium shows that the colony-forming unit (CFU) total number was 100 fold higher than on CTEA. The thermophilic phase is determined at 30 day for both mixtures A and B. Nevertheless this stage is limited only at 22 and 30 days, respectively for mixture A and B on CTEA medium, which indicate a similar temperature profile at versus time of co-composting.

The results suggest that the GSM medium approach can be used for monitoring the microbial cultivable presence. However, CTEA act as a natural selective medium to enumerate the indigenous functional microflora. This technique was successful in assessing the process evolution and determination of a real succession thermophilic and maturation co-composting stages.

## Introduction

The conversion of sewage sludge into organic fertilizer, play a significant role to improve soil fertility by influencing the physical, chemical and biological properties. The process of aerobic sewage sludge composting can be separated into principal distinct stages, stabilization and maturation one. However, the microorganisms that populate substrates during composting reflect the evolution and the performance of the process [1]. The development of thermophilic bacteria could determine the stabilization stage characterized by a significant temperature rises (up to 50°C). After, the temperature gradually decreases to ambient conditions which determine the second composting phase (maturation). Many different groups of microorganisms found in composted raw matter. Several techniques have been explored to assess microbiological activity in composting systems [2,3]. However, the relationship between compost stability and functional microflora is not well understood. Current knowledge of the compost microbial community is based on different approaches such as direct analysis of phospholipid fatty acid patterns (PLFA) [4] or molecular techniques [5]. Klammer *et al*. [6] differentiated microbial communities of mature compost originating from various organic wastes, using denaturing gradient gel electrophoresis (DGGE). Drennan and DiStelfano [7] measured oxygen uptake rates, oxygen consumption, temperature, volatile solids. Also, Solvita Maturity Index is used to determine microbial activity of curing municipal solid waste in an insulated reactor. However, the traditional microbiological approach is still effectual [3]. Several authors, Ishii and Takii [8], Lei and Vander Gheynst [9] show some restrictions due to the inherent selectivity of culture media. Therefore, any approach focused only on selective media of only functional microflora which able to degrade a composted substrates.

This study was directed to investigate the functional indigenous microflora dynamics, and their relation to play a significant changes in the physical and chemical parameters of the co-composting substrates, and that, in turn, their role to determination the real principals succession stages of process evolution at versus time of co-composting, by using traditional culture approaches, growth standard medium (GSM), and a Co-composing Time Extract Agar (CTEA) as a selective medium.

## Materials and methods

### Co-composting trials

Co-composting trials were conducted for six months on a composting platform located in the plant nursery of Marrakesh. Two trials with different proportions sewage sludge/palm waste were followed:

- Mixture A: 1/3 sludge + date palm tree waste 2/3, total volume: 4 m^3^.
- Mixture B: 1/2 sludge + date palm tree waste 1/2, total volume: 4 m^3^.

Each mixture was carefully homogenized, moisture was adjusted to 60% (optimal value for composting), and then the mixtures were windrowed. Windrows were turned over by hand with a weekly frequency to aerate the mixture. Homogenous samples were taken at T_0_ (first day of co-composting) and after airing (aerating the mixture). Homogeneous samples (1 kg) were obtained by careful mixing of several sub samples taken at different points (height and length) of the windrow, then quartering. The samples were kept at −20°C before analysis.

### Physico-chemical analyses

The temperature was measured every day at different levels (height and length) of the windrow using sensors with data memory (PH0700115 model 1.20, Ector-Traceability software, ECTOR France). The samples were dried out at 105°C. The pH was measured in an aqueous extract of the compost at room temperature (1 g/10 ml of distilled water) according to NF ISO 10390. Total organic carbon and Ash content were calculated after calcination in a muffle furnace at 600°C for 6 h. Total Kjeldahl nitrogen (TKN) was assayed in 0.5 g samples by using classical Kjeldahl procedure, by steam distillation according to AFNOR T90-1110 standard.

### Biological analysis

Indigenous microflora was enumerated, by using growth standard nutrient agar and (CTEA) media. Samples of each co-composting time ( 0, 15, 22, 30, 60, and 180 days) were first mixed, suspended in sterile distilled water (10 g in 100 ml) homogenized by vortexing and finally treated 10 to 15 min by sonication according to [10]. All treated samples were serially diluted up to 10^−9^ and cultivable microbial flora was enumerated by pour plating and spreading 0.1 ml from 10^−4^, up to 10^−9^ of Standard Medium (GSM) (Nutrient agar), and of Co-composting Time Extracts Agar (CTEA) prepared as follow: One litre of distilled water and 35 g of co-composting time were mixed overnight. After filtration and sterilization at 120°C for 15 min, agar (15g) was added to the collected filtrate. The pH was adjusted to recorded pH for the co-composting time before sterilization. For each co-composting time (0, 15, 22, 30, 60 and 180 days), Three replicate were made and the plates were incubated at 28°C for enumeration of total mesophilic microflora and 45°C for total thermophilic.

### Statistical analysis

The results are presented in the form of averages ± SEM. The comparison of the averages is made by ANOVA (SPSS Win version 10). The differences are considered significant at p <5%.

## Results and discussion

### Physical-chemical characterization

#### Temperature evolution during co-composting process

**Figure 1:**
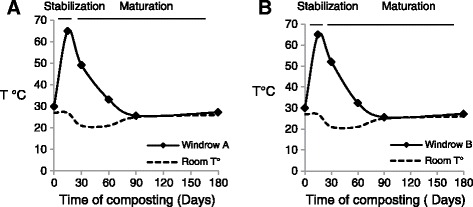
**Temperature versus time during the co-composting process for two mixtures A (A) and B (B), according to** **[** 1 **]** **.**

The microbial community succession changed markedly as composting process progressed; particular correlations could be drawn between the biological and physico-chemical parameters especially temperature. In the present study (Figure 1), the temperature indicates a typical composting pattern characterized by two major phases. Thermophilic phase (stabilization phase) characterized by a rise in temperature which peaked its maximum value at 65°C at 15 day of the process. The heating stage is due to intense microbial activity resulting from the degradation of the simple molecules present in the substrate [1,11]. The maturation phase which characterised by a decrease of temperature, due to the exhaustion of easily metabolisable organic compounds and the availability of recalcitrant substrates (e.g. lignin, cellulose) [12]. The principal characteristics of composting products are presented in (Table 1) the final products presented a C/N ratio around 10, NH_4_^+^/NO_3_^−^ ≤ 1, which provide information about the importance of organic matter oxidation and significant degree of degradability of substrate. As indicated in the literature, these values confirm the maturity of compost [1,11].

**Table 1:** **Physico-chemical parameters during co-composting process for mixture A and B, according to** [1]

**Mixture**	**Time of co-composting (months)**	%TKN*	**Moisture**	**NH**_**4**_^**+**^ **/NO**_**3**_^**−**^	**C/N**	DEC (%)*	**pH**
A	0	1.31 ± 1.2	58.83	13.75	26.2	-	6.34 ± 0.03
	6	2.18 ± 1.1	66	0.12	10.09	40.07 ± 1,1	6.79 ± 0.06
B	0	1.28 ± 1.09	60.97	15.6	27.4	-	6.04 ± 0.28
	6	2.28 ± 1.21	66	0.14	10.08	40 ± 1.49	7.03 ± 0.08

(*): Results expressed per unit weight dry matter;TKN = Total Kjeldahl Nitrogen; DEC = Decompositionrate.

### Indigenous microflora abundances during co-composting

**Figure 2:**
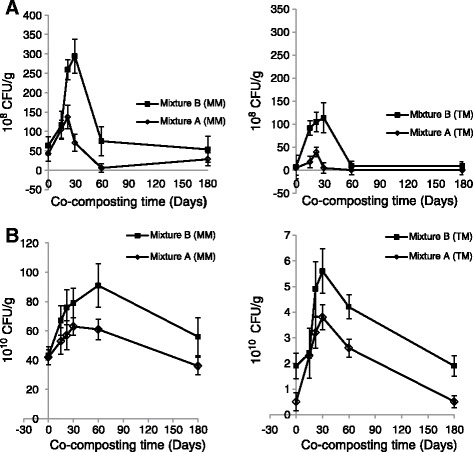
**Mesophilic and thermophilic microflora evolution during co-composting of mixture A and B by using CTEA and GSM media. A** Mesophilic and thermophilic microflora evolution in CTEA medium. **B** Mesophilic and thermophilic microflora evolution in GSM medium. *TM: Thermophilic Microflora, MM: Mesophilic Microflora. CTEA: Co-compost Time Extract Agar. GSM: Growth Standard Medium. Microflora = moulds, yeast, bacteria and actinobacteria.

The succession of the different microbial populations is illustrated in (Figure 2A, 2B). Particular difference has been shown, between microorganisms dynamic in two culture media (CTEA, and GSM). Gurtner *et al*. [13] illustrated that no similar microorganisms could be detected by both culture dependent and molecular methods.

Total mesophilic and thermophilic microflora of activated sludge and date palm waste composting, show a same development appearance (profile) at versus time of co-composting on the CTEA medium (Figure 2A). However, the different profiles have been shown in GSM medium (Figure 2B). The total mesophilic and thermophilic microflora in GSM (Figure 2B) is 100 fold higher than the corresponding mesophilic and thermophilic groups in CTEA medium. This is due to the possibility of development of cultivable functional and nonfunctional microflora on GSM medium. However, the CTEA could be act as a selective medium for functional microorganism which could use the co-composting substrates as source of their growth, and contribute to the transformation process at the composting time pH and the temperature.

### Microbial communities succession during co-composting

In this study, both mesophilic and thermophilic microflora evolution show a peak during thermophilic stage for mixtures A and B. However, the significant difference has been shown between CTEA and GSM media. The total mesophilic microflora increases from 43 × 10^8^ CFU/g to 137 × 10^8^ CFU/g on 22 day (thermophilic phase), and from 63 × 10^8^ CFU/g to 294× 10^8^ CFU/g on 30 day, respectively for mixture A and B on CTEA medium (Figure 2A). That is closely correlated with thermophilic phase determined by temperature evolution during co-composting process (Figure 1). As reported in the literature, the thermophilic stage varies from a few days to several months [14]. Nevertheless, we noted that mesophilic microflora on GSM medium increases from 42 × 10^10^ CFU/g to 63 × 10^10^ CFU/g on 30 day, and from 43 × 10^10^ CFU/g to 91 × 10^10^ CFU/g on 60 day of co-composting, respectively for mixtures A and B. The thermophilic stage which is determined by mesophilic microflora evolution is not correlated with temperature evolution during co-composting. These results confirm that CTEA is a selective medium for functional microflora which could give information of the real determination of both co-composting stages.

The high mesophilic microflora value at thermophilic phase of co-composting which spread until 30 and 60 days respectively for CTEA and GSM media (Figure 2A, 2B), is due to the abundance rate of mesophilic and thermotolerant microflora in the composted substrates. These findings are coinciding with those of [2] who revealed, on nutrient agar, a significant increase of aerobic heterotroph bacteria in the thermophilic phase. Chroni et al. [3] indicated that total mesophilic bacteria on nutrient agar increases on day 57 of the process. The total thermophilic grew fast on day 59 when the highest temperature for about 5 weeks was recorded. That could explain that GSM approach gives information also on non functional microorganisms which are not appropriate to determine the real co-composting stage.

The evolution of thermophilic microflora in CTEA medium varies in the range from 5.4 × 10^8^ to 40 × 10^8^ CFU/g, and from 7.7 × 10^8^ to 114 × 10^8^ CFU/g respectively for mixtures A and B. On the GSM medium, from 0.5 × 10^10^ CFU/g to 3.8 × 10^10^ CFU/g and from 1.9 × 10^10^ CFU/g to 5.6 × 10^10^ CFU/g respectively for mixtures A and B. The high recorded values on 30 day for both culture media followed a temperature rise during thermophilic phase (Figures 1 and 2A, 2B), could be a result of a growth and activity of thermophilic and thermotolerant microflora during co-composting. Thereafter, the thermophilic microflora shows a significant decrease after 30 day of co-composting. For mixture A in CTEA medium, the thermophilic microflora growth is limited at 22 day (Figure 2A). That could be attributed to the fast thermophilic microflora change in mixture A. Haug [15] showed that the degradation of easy metabolisable molecules by mesophilic microflora at composting beginning stage leads to a significant rise of temperature which in turn leads to change in the microbial community structure.

All mesophilic and thermophilic microflora abundances declined at second stage of co-composting (maturation stage). This is due to the restricted conditions particularly the lost of easy metabolisable substrates. The decrease of temperature affects especially the thermophilic microflora development [16]. Sidhu *et al*. [17] showed that the overall population of bacteria declined with the progress of the composting process. This is not unexpected as the concentration of readily available nutrients, moisture content, and temperature of compost pile declined with maturity.

## Conclusion

This study investigates the microbiological succession during six months of aerobic composting of active sludge and date palm waste using two culture media. The thermophilic microflora varies in the range from 5.4 × 10^8^ to 40× 10^8^ CFU/g at 22 day on CTEA medium, and from 7.7 × 10^8^ to 114 × 10^8^ CFU/g at 30 day respectively for mixtures A and B. However, on GSM medium, the CFU number varies from 0.5 × 10^10^ CFU/g to 3.8 × 10^10^ CFU/g and from 1.9 × 10^10^ CFU/g to 5.6 × 10^10^ CFU/g at 30 day, respectively for mixture A and B. CTEA medium adjusted to recorded pH of co-composting time, offer qualitative information on the microbial co-composting succession and on the evolution of the co-composting process. However, the GSM medium indicates only the microbial presence. There was still insufficient evidence to ensure that the microbial community composition is able to transform the co-composting matters. That is one disadvantage of using of growth standard medium for mesophilic and thermophilic microflora enumeration.
